# Irisin and Myonectin Regulation in the Insulin Resistant Muscle: Implications to Adipose Tissue: Muscle Crosstalk

**DOI:** 10.1155/2015/359159

**Published:** 2015-05-05

**Authors:** Luis Gamas, Paulo Matafome, Raquel Seiça

**Affiliations:** ^1^Laboratory of Physiology, Institute for Biomedical Imaging and Life Sciences (IBILI), Faculty of Medicine, University of Coimbra, 3000-548 Coimbra, Portugal; ^2^Department of Complementary Sciences, Coimbra Health School (ESTeSC), Instituto Politécnico de Coimbra, 3040 Coimbra, Portugal

## Abstract

Myokines are peptides produced and secreted by the skeletal muscle, with autocrine, paracrine, and endocrine actions. Many of them are overexpressed during physical exercise and appear to contribute to the benefits of exercise to metabolic homeostasis. Irisin, resulting from the cleavage of the membrane protein FNDC5, was shown to induce adipocyte browning, with increased lipid oxidation and thermogenesis. Myonectin was only recently discovered and initial studies revealed a role in fatty acid uptake and oxidation in adipose tissue and liver. However, the mechanisms of their regulation by exercise are not entirely established. Impaired secretion and action of myokines, such as irisin and myonectin, may have a role in the establishment of insulin resistance. On the other hand, several studies have shown that insulin resistance in the skeletal muscle may change myokines expression and secretion. This may have consequences on lipid and glucose metabolism in adipose tissue and lead to a vicious cycle between impaired myokines production and insulin resistance. This review summarizes the current knowledge about the influence of skeletal muscle insulin resistance on the secretion of irisin and myonectin, as well as its impact on adipose tissue metabolism.

## 1. Introduction

Diabetes mellitus is one of the most prevalent pathologies worldwide affecting 8% of the population [1]. Although diabetes mellitus is generally characterized by chronic hyperglycemia due to inappropriate pancreatic function, type 2 diabetes also results from insulin resistance, mainly in skeletal muscle, liver, and adipose tissue [2]. Insulin resistance typically develops in conditions of excessive fat mass, leading to a compensatory increase of insulin secretion by pancreatic *β* cells and hyperinsulinemia. However, when *β* cells are no longer able to compensate for insulin resistance, hyperglycemia develops [3].

Insulin is known to act through a tyrosine kinase receptor, which phosphorylates the insulin receptor substrates (IRS-1 and IRS-2), leading to successive PI_3_K and protein kinase B (PKB)/Akt activation [4, 5]. The main postprandial actions of insulin include the translocation of GLUT4 to the membrane of myocytes and adipocytes, activation of glucokinase in hepatocytes, and inhibition of lipolysis and gluconeogenesis [1].

Such processes are totally or partially compromised in type 2 diabetes, due to the development of insulin resistance, which is mainly based on the desensitization of the insulin receptor and impaired phosphorylation of its substrates. The skeletal muscle is particularly important in insulin resistance, as it uptakes most of the postprandial glucose. As several myokines were shown to be produced as a consequence of muscle glucose uptake, it is expected that insulin resistance could change myokines secretion. Also, myokines secretion was shown to be changed by physical exercise. Since the development of insulin resistance completely changes myocytes metabolism, the secretion of myokines during the exercise may also be compromised.

In the recent years, irisin and myonectin rose as important myokines secreted in response to physical exercise and also dietary glucose and fatty acids, promoting glucose and fatty acid uptake and oxidation in liver and adipose tissue and, in the case of irisin, thermogenesis. This review will summarize the current knowledge about the impact of muscle insulin resistance in irisin and myonectin secretion, focusing on the possible metabolic consequences in adipose tissue.

## 2. Muscle Insulin Resistance in Obesity: Lipotoxicity and Inflammation

One of the mechanisms firstly shown to cause insulin resistance was the accumulation of secondary products of lipid metabolism, such as diacylglycerol, ceramides, and long-chain acetyl coenzyme A. The accumulation of such products in myocytes was shown to activate serine/threonine kinases like c-jun N-terminal kinase (JNK), I*κ*B kinase (IKK), and protein kinase C (PKC), conducting to serine phosphorylation and consequent inactivation of the insulin receptor and its substrates [4–7].

Insulin resistance was also shown to be correlated with impaired lipid oxidation in mitochondria. This was shown to be caused by impaired mitochondria biogenesis and also to decreased levels of peroxisome proliferator-activated receptor gamma coactivator 1-*α* (PGC-1*α*) and 1-*β* (PGC-1*β*) [4, 5]. Thus, decreased mitochondria number and impairment of lipid oxidation mechanisms further contribute to the accumulation of secondary lipid metabolites and to the inactivation of insulin signaling.

Moreover, increased reactive oxygen species (ROS), consequent to lipotoxicity and impaired mitochondrial function, are also thought to cause insulin resistance [4, 8]. The imbalance between oxidant and antioxidant compounds leads to the activation of stress pathways, such as JNK, IKK, and p38-mitogen-activated protein kinase (p38-MAPK). As mentioned above, these serine/threonine kinases directly inhibit the insulin receptor pathway. Moreover, ROS also inhibit mitochondria function, leading to further intracellular fatty acid accumulation, thus creating a vicious cycle of lipotoxicity and insulin resistance (reviewed by [4]).

As a result of the activation of stress pathways in response to lipid metabolites and oxidative stress, cellular inflammatory mechanisms are activated. JNK and PKC lead to IKK activation and thus to NF-*κ*B translocation to the nucleus and, consequently, to the expression of proinflammatory cytokines, such as tumor necrosis factor- (TNF-) *α* and interleukin- (IL-) 6 [4, 8–12]. Also, NF-kB activation favors the expression of chemoattractant factors, such as the monocyte chemoattractant protein- (MCP-) 1 and the migration inhibitory factor (MIF), which recruit and increase the permanence of macrophages in the tissues, especially in adipose tissue and liver. Such macrophages are also important sources of proinflammatory cytokines [4, 13]. Like intracellular lipids, extracellular lipids are also able to activate inflammatory pathways. Extracellular free fatty acids may be recognized by the innate receptor toll-like- (TLR-) 4. Along with proinflammatory cytokines, such mechanisms trigger inflammatory signals, which create an inflammatory feedback (reviewed by [13]). Thus, obesity is associated with a low-grade inflammation, which derives from excessive lipid deposition and inhibits insulin signaling, namely, in muscle, liver, and adipose tissues.

## 3. Myokines: Crosstalk Muscle—Adipose Tissue and Sedentarism-Associated Diseases

Hormonal function of the muscle has only been described in the recent years, based on the identification of peptides released under several circumstances and acting in a systemic manner. Such peptides have been commonly named as myokines and raised the importance of the muscle hormonal function. Most of them are released in response to muscle contraction during physical exercise but also in response to nutritional changes [14–16]. Although the muscle responds directly to insulin increasing GLUT4 translocation and glycogen synthesis, myokines also influence the whole body metabolism of glucose and lipids and the energy balance, as they were shown to act on adipose tissue, liver, pancreas, and intestine [15, 17]. Several myokines were recently identified, including interleukins (IL-6, IL-8, IL-7, and IL-15), irisin, myostatin, myonectin, the brain-derived neurotrophic factor (BDNF), insulin-like growth factor-1 (IGF-1), leukemia inhibitory factor (LIF), and follistatin-like-protein-1 (FSTL-1) [15].

Regular physical activity has a well-known protective role on several sedentarism-associated pathological conditions, such as insulin resistance, obesity, atherosclerosis, type 2 diabetes, neurodegenerative disorders, and even several types of cancer like breast and colon [18, 19]. Physical inactivity favors the accumulation of visceral adipose tissue and the resulting low-grade inflammation, which is known to be associated with such pathologies. Thus, physical exercise has direct anti-inflammatory effects due to the decrease of adipose tissue accumulation. However, such events alone do not explain the beneficial effects of exercise. Some myokines were shown to be released during physical exercise and to mediate some of its valuable outcomes. In particular, irisin and myonectin were shown to act on adipose tissue and control glucose and lipid metabolism.

### 3.1. Adipose Tissue Browning

Brown adipose tissue (BAT) is known to have an increased ability to oxidize lipids and produce heat, due to the high number of mitochondria and the expression of the uncoupling protein- (UCP-) 1. BAT is activated under low temperature conditions and is very sensitive to insulin, dramatically increasing its irrigation and glucose uptake [20]. BAT was believed to be almost inexistent in adults, but this idea has recently changed. A metabolically active BAT in the cervical and thoracic regions was identified by Cypess and coworkers using PET-^18^F-FDG and immunohistochemistry. They showed multilocular adipocytes and positive UCP-1 staining, both of them, features of BAT [21]. Furthermore, it has more recently been shown that BAT activity is correlated with increased energy expenditure and weight and fat mass loss. Moreover, obese individuals were shown to have decreased BAT activity [22, 23]. Recently, Wu and coworkers characterized a third type of adipocytes, the beige or brite (brown + white) adipocytes [24]. According to the authors, this type of adipocytes has intermediate characteristics between the white and the brown ones. They demonstrated that brite adipocytes have UCP-1 expression and the ability to increase thermogenesis when stimulated by cold or by *β*3-adrenergic activators [24]. The process of brite adipocytes stimulation is known as browning, which consist in the development of brite adipocytes within white adipose tissue depots, especially subcutaneous ones, probably from the same precursor cell of the white adipocytes. The same authors hypothesized that the induction of browning in white adipose tissue could be an effective strategy to increase lipid metabolism and improve obesity and type 2 diabetes.

### 3.2. Irisin/FNDC5

**Figure 1:**
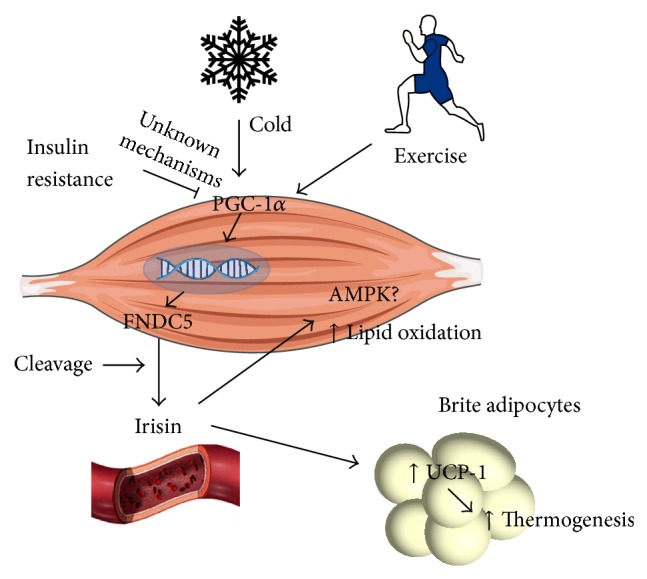
Stimulation of irisin secretion by cold and exercise. Irisin secretion results from PGC-1a activation, which leads to FNDC5 expression. FNDC5 present in the membrane is in turn cleaved and irisin is secreted to the blood, acting on the adipose tissue in order to stimulate the formation of brite adipocytes and UCP-1 expression. Irisin also seems to induce AMPK phosphorylation in the skeletal muscle itself. AMPK: AMP-activated protein kinase; FNDC5: fibronectin type-III domain containing protein 5; PGC-1*α*: peroxisome proliferator-activated receptor gamma coactivator 1-*α*; UCP-1: uncoupling protein-1.

Irisin was only recently characterized as a cleavage product of the transmembrane protein fibronectin type-III domain containing protein 5 (FNDC5) [25]. In 2012, Boström et al. demonstrated that physical exercise was able to increase the skeletal muscle expression of several genes involved in energy expenditure and particularly in glucose and lipid metabolism. Among such genes was FNDC5, located on locus 1p35.1, which was shown to be expressed as a consequence of PGC-1*α* activation. Moreover, the 32 kD FNDC5 protein is then cleaved and released in the circulation in the form of irisin, a 22 kD protein (Figure 1) [25, 26]. Authors showed increased circulating levels of irisin in animals and humans subjected to an exercise program. Authors also described that irisin was acting on subcutaneous adipose tissue in order to induce browning, that is, increased thermogenesis and energy expenditure. Using a protocol of FNDC5 overexpression through viral vectors, they observed decreased fat mass and improved glucose tolerance in diet-induced obese and insulin resistant mice [25]. In addition, Wu and coworkers described that irisin strongly increased a browning response in brite but not in brown adipocytes [24], a result which has been replicated in at least two later studies [27, 28].

Accordingly, other studies showed no increase in BAT activity after physical activity, suggesting that irisin may act mainly in brite but not in brown adipocytes [29, 30]. A study by Wrann et al. interestingly found that FNDC5 in mice is preferably expressed by oxidative muscles [31]. A recent study, which assessed the effects of fenofibrate, a PPAR-*α* agonist and, therefore, an activator of PGC-1*α*, found an increase of serum irisin levels and UCP-1 expression in mice [32]. Thus, by increasing adipose tissue browning, irisin administration could be an effective therapeutic strategy to improve insulin signaling, mimicking exercise effects [33].

Despite the promising results, the regulation and the physiological actions of irisin are not yet fully understood and some studies even questioned the initial findings, showing disappointing results. The serum concentrations detected differ in various studies, ranging from 0.04 ng/mL to 2.158 ng/mL, possibly due to the different enzyme immunoassay kits used. It is important to note that none of the antibodies have been tested for cross-reactions with other serum proteins [14]. Although many studies have described the effects of irisin on subcutaneous adipose tissue, at least a couple of studies have reported its browning-promoting effects of visceral adipose tissue [34, 35]. In the case of the study of Roca-Rivada and coworkers, authors showed visceral adipose tissue browning of Sprague-Dawley rats in response to irisin but not in subcutaneous depots [35]. Moreover, several studies performed in human subjects have not confirmed the role of irisin in promoting browning. A study performed by Norheim and colleagues showed no effect of a 12-week training program on white adipose tissue browning (normal levels of UCP-1), on myocyte FNDC5 mRNA levels nor on circulating irisin levels [36]. Interestingly, circulating irisin levels were decreased after the training program but showed a superior increase after acute exercise. After the training program, authors also observed increased FNDC5 levels, suggesting that acute exercise may increase its cleavage and thus the circulating irisin, returning to normal levels between exercise periods. Despite this, other studies performed in humans reported no elevation of FNDC5 and most of them showed no changes in circulating irisin after acute or chronic exercise [37–47]. However, recent studies have shown new data about this myokine. Lee et al. compared the effects of maximal and submaximal exercise sessions on serum irisin levels of healthy subjects, showing higher levels in the latter, suggesting that endurance exercise is more efficient than short term high intensity exercise in inducing irisin secretion. Moreover, the authors found a cold-induced raise serum irisin levels, which was highest in subjects reporting shivering, further pointing out a muscle contraction-dependent mechanism [27]. Other studies have confirmed a positive correlation between acute exercise sessions and circulating irisin [48–51]. Tsuchiya et al. pointed out that exercise intensity is positively correlated with the serum irisin raise, independently of energy consumption [51]. It is important to note that all of these studies used an aerobic exercise program, therefore, soliciting preferably oxidative muscle fibers. Interestingly, chronic exercise training does not seem to be correlated with higher serum irisin levels [36, 50, 52], which suggests that an adaptive mechanism is present, making physically active individuals less dependent of irisin for lipid metabolism or more sensitive to irisin action.

Most of the* in vitro* studies using cultured human myocytes revealed no increases in FNDC5 expression after stimulation with ionomycin (calcium raiser) or electric stimuli [36, 38, 40]. Interestingly, in all of these studies PGC-1*α* levels were increased, questioning its involvement in FNDC5 and irisin expression and secretion. However, a recent study from Huh and colleagues showed increased FNDC5 expression in primary human skeletal muscle cells after ionomycin and forskolin treatment [48]. Moreover, in the study of Lee et al., authors showed browning and heat production in human adipocytes incubated with FNDC5 [27]. The molecular mechanisms of irisin expression, secretion, and actions on adipose tissue remain partially unknown and further studies are needed (see reviews at [53, 54]).

Apart from the browning of adipose tissue, irisin also seems to act on the skeletal muscle itself. Two studieshave demonstrated that irisin induces AMPK phosphorylation and glucose uptake in cultured human skeletal muscle cells [48, 55]. In one of them, irisin was also shown to induce fatty acid uptake [48]. Vaughan et al. assessed the effects of irisin on cultured murine myocytes and observed an increase of oxidative metabolism, PGC-1*α*, and even irisin itself [56].

### 3.3. Myonectin

**Figure 2:**
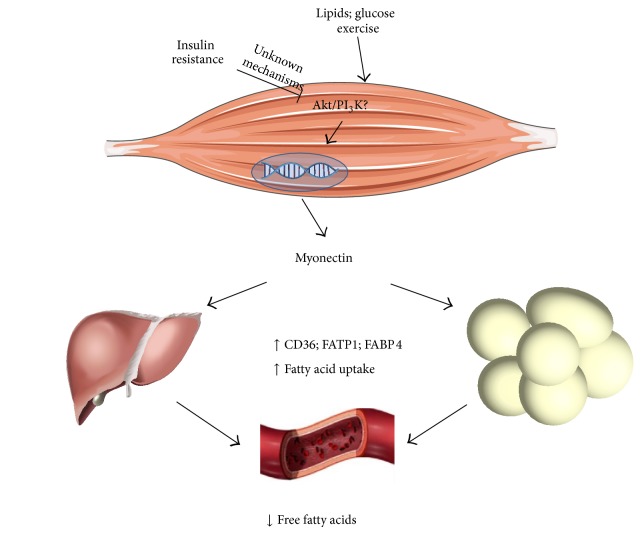
Stimulation of myonectin secretion by exercise, lipids, and glucose. The activation of unknown myocyte mediators by exercise and nutrients, which may include the Akt/PI_3_K pathway increases myonectin secretion. Myonectin increases the expression of fatty acids transporter and binding proteins in adipose tissue and liver, which promotes fatty acid uptake and storage. FABP: fatty acid binding protein; FATP: fatty acid transporter protein; PI_3_K: phosphatidylinositol-3-kinase.

Myonectin is also known as C1q/TNF-related protein isoform 15 (CTRP15), whose gene is located on locus 2q37.3. This myokine was discovered very recently by Seldin and coworkers. According to the authors, myonectin expression is stimulated by two main factors: exercise and nutrients (Figure 2) [16]. Fasting circulating myonectin levels were shown to be low and to increase 2 hours after the intake of glucose or lipids. Furthermore, ionomycin-induced increase of intramyocyte calcium concentration and a 2-week program of exercise in mice were shown to significantly increase myonectin secretion [16]. However, it is not clear whether elevation of myonectin levels is due to the exercise itself or to the intake of glucose and lipids following the exercise program.

Myonectin functions are apparently related to lipid metabolism, acting in order to decrease plasma-free fatty acids levels through the stimulation of their uptake in adipose tissue and liver. These effects appear to be mediated by an increase of scavenger and transporter proteins, such as CD36, fatty acid transporter protein- (FATP-) 1, and fatty acid binding protein- (FABP-) 4 (Figure 2) (reviewed by [57]). On the other hand, myonectin has no effects on adipocyte lipolysis and glucose homeostasis [16]. Based on such observations, authors proposed that myonectin could be a nutrient-sensing myokine, informing other tissues about the nutrient status and promoting their uptake and storage. Also, the same authors observed that myonectin acts on the liver in order to inhibit autophagy through the activation of the PI_3_K/Akt/mTOR pathway, supporting the idea that it simultaneously regulates and is regulated by the nutritional state [58]. However, the pathways conducing to myonectin muscle expression in the postprandial period are unknown and future studies will be necessary to address these questions.

### 3.4. Irisin and Myonectin Regulation by Insulin Resistance

Irisin and myonectin are likely to be involved in lipid and glucose metabolism and thus may prevent the development of insulin resistance. However, their secretion may also be affected by the development of muscle insulin resistance. Since irisin and myonectin were shown to act in the adipose tissue, their dysregulation may affect the crosstalk between the tissues and further contribute to insulin resistance and to impaired glucose and lipid metabolism.

Several studies have addressed the association between decreased serum irisin levels and insulin resistance or diabetes. Many studies found lower circulating irisin levels in type 2 diabetic patients [59–65] and others found a negative correlation with fasting glucose levels [66] and HbA1c [64, 66]. Preliminary data from our laboratory also showed decreased circulating irisin levels in the nonobese type 2 diabetic Goto-Kakizaki rats (data not published). This suggests that irisin levels may be affected by the development of insulin resistance.

However, the regulation of irisin in obesity (without glucose dysmetabolism) may be quite different. A recent work performed by Pardo and colleagues showed increased circulating irisin levels in extremely obese women. Irisin levels were correlated with body mass index (BMI) and fat mass and authors described adipose tissue as the main source of circulating irisin in such patients [67]. Other authors have also found a positive correlation between BMI, fat mass, and serum irisin levels [52, 68, 69]. Authors hypothesized that obesity may be linked with the development of irisin resistance, similarly to what is observed for insulin and leptin. If confirmed, promotion of irisin secretion through pharmacological strategies or use of irisin as a therapeutic agent could both prove ineffective. On the other hand, the raise in serum irisin levels in obesity may be a compensatory mechanism, in order to induce lipid metabolism.

Irisin has also been tested as a predictor of adverse outcomes related with the metabolic syndrome. In obese individuals with other cardiovascular risk factors, higher serum irisin levels were found to be correlated with lower HDL cholesterol [70]. Irisin has also been found to be positively correlated with VLDL, triglycerides, and total cholesterol [52]. Since all of these represent cardiovascular risk factors, it might seem logical that higher serum irisin levels would predict unwilling outcomes. However, a study performed in diabetic patients showed that those with macrovascular disease typically presented lower serum irisin levels. Lower serum irisin levels were an independent predictor of macrovascular disease in type 2 diabetic patients [60]. Such observations support the idea of irisin downregulation by muscle insulin resistance, correlating negatively with glucose dysmetabolism.

Myonectin effects on lipid and glucose metabolism opened promising therapeutic possibilities. However, the molecular mechanisms of myonectin expression, secretion, and action have not been yet identified. In the original study of Seldin et al. identifying myonectin, authors showed decreased myonectin levels in high-fat diet-fed mice, resulting from decreased mRNA levels in skeletal muscle [16]. Such results suggested that decreased circulating myonectin levels could contribute to decreased free fatty acids uptake in adipose tissue and thus to the elevation of their circulating levels and to ectopic accumulation in other tissues. However, a recent study by Peterson and colleagues showed increased circulating myonectin in obese Zucker rats [71]. Given that Zucker rats are genetically defective in the leptin receptor and consistently present hyperleptinemia, such effects may be caused by chronic myocyte stimulation by leptin. In fact, Rodríguez and colleagues recently showed that leptin acts on the myocyte in order to induce the expression of the myonectin gene [72]. The impact of hyperleptinemia in conditions of receptor desensitization needs to be addressed in the future. On the other hand, serum myonectin levels were observed to be unchanged in calorie restricted rats [73].

Myonectin regulation by exercise is also contradictory, taking into account the studies of Seldin and Peterson. Peterson et al. found decreased myonectin mRNA but increased intramuscular myonectin levels on Zucker rats after exercise [71]. A pitfall of this study was the absence of myonectin determination in serum samples, even though authors have hypothesized that myonectin circulating levels could be correlated with intramuscular levels. Different regulation of myonectin by exercise programs, in the previous studies, is probably related to the experimental animal models, specifically to differences in leptin action. Altogether, the results currently available suggest that myonectin expression may be regulated by leptin, but its secretion may be triggered by the nutritional state, as demonstrated by increased secretion after physical exercise. Decreased myonectin expression in the study by Peterson may reflect decreased leptin levels after the exercise program, even though authors did not measure them.

Currently, the studies focusing on the effects of insulin resistance in myonectin levels are scarce. Recently, Yang et al. showed that cultured mice myotubes with palmitate-induced insulin resistance had lower levels of myonectin and FNDC5 expression. Authors observed decreased Akt activation and suggested that the expression of myonectin gene may be increased by PI3K and decreased by the p38 MAPK. Palmitate was observed to decrease PI3K and to increase p38 expression, leading to decreased myonectin expression [74].

## 4. Conclusions and Future Perspectives

The skeletal muscle is one of the main targets of insulin and one of the first tissues to develop insulin resistance, commonly in conditions of obesity and dysregulated lipid metabolism. Given its endocrine function of secreting many factors that control glucose and lipid metabolism in response to exercise and alterations of the nutritional state, it is expectable that the development of insulin resistance may somehow change the skeletal muscle secretome. Such changes may have impact on organ tissues also involved in glucose and lipid homeostasis like the adipose tissue. However, it is not clear whether impaired myokine secretion occurs as a result of insulin resistance or, in turn, as a parallel event. The understanding of myokine physiology and pathophysiology may open new windows in order to pharmacologically mimic exercise effects on obese and type 2 diabetic patients. In the study performed by Raschid et al., fenofibrate, besides increasing circulating irisin levels and inducing adipose tissue browning in obese mice, also decreased adiposity and glucose intolerance. Such results open new promising possibilities, even if they need to be confirmed in humans. Myokine regulation in such diseases is not known, as the secretion of some of them may be inhibited, as observed for adiponectin, or in turn tissues may become resistant to their actions, as observed for insulin and leptin. Such differences will certainly influence its therapeutic usefulness in the future. Future studies should be focused on the molecular mechanisms underlying irisin and myonectin expression, secretion, and action in order to identify new therapeutic targets that could mimic exercise effects on lipid and glucose homeostasis, improving lipid storage in adipocytes and insulin sensitivity in the liver, the adipose tissue, and the muscle itself.
